# Protective effects of compatible solute ectoine against ethanol-induced toxic alterations in *Daphnia magna*

**DOI:** 10.1007/s00360-018-1165-2

**Published:** 2018-06-08

**Authors:** Adam Bownik, Brygida Ślaska, Aleksandra Szabelak

**Affiliations:** Institute of Biological Basis of Animal Production, University of Life Sciences in Lublin, 20-950 Lublin, Poland

**Keywords:** Ectoine, Ethanol, *Daphnia magna*, Protective effects

## Abstract

Ectoine (ECT) is a compatible solute synthesized mostly by halophilic microorganisms subjected to various stressful factors. Its protective properties in bacteria and some populations of isolated cells subjected to different stressors are reported; however, little is known on its effects against a commonly used compound, ethanol (ETH). The purpose of our study was to determine the effects of ETH alone (at 20 and 60 g/L) and in the combination with various concentrations of ECT (5, 10, and 25 mg/L) at various times of exposure on behavioural, physiological, and biochemical parameters of a model invertebrate *Daphnia magna*. In the present study, we determined the following parameters: immobilisation, heart rate, thoracic limb movement, catalase (CAT) activity, and nitric oxide species (NO_*x*_) level. Our study revealed that both concentrations of ETH alone induced immobilisation and decrease of swimming velocity, heart rate, and thoracic limb activity; however, catalase activity and NO_*x*_ levels were increased. On the other hand, the animals exposed to the combinations of ETH + ECT showed a reduced immobilisation and alleviated inhibition of heart rate and thoracic limb activity, lower increase of CAT activity, and NO_*x*_ level when compared to the crustaceans subjected to ETH alone. The most distinct alleviation of toxic effects was noted in the combinations in which the highest concentration of ECT were used. The results suggest that ETH may induce oxidative stress in daphnids and attenuating effects of ECT probably result from its antioxidative properties.

## Introduction

Ethanol (ETH) is an organic compound used as the main ingredient of alcoholic beverages and as a solvent for perfumes, various drugs, and as a fuel additive in some countries (Zucotti and Fabiano 2011). Some results indicate that ETH is a neurotoxic compound with a potential to induce neurodegenerative diseases resulting from oxidative stress (Song et al. 2014) and it also may act as a teratogen (Randall 1987; Kietzman et al. 2014). Since ETH is used as a fuel additive particularly in the United States and Brazil, the risk of contamination of surface water has increased (Barros 2013). It is generally accepted that the breakdown of ETH in surface waters by biological and chemical processes results in the consumption of significant quantities of oxygen which may affect aquatic life, especially fish (Poltak and Grumet 2001; Weaver 2009). However, little is known on direct toxic effects of ethanol itself on behavioural, physiological, and biochemical parameters of aquatic invertebrates.

Some organisms produce compatible solutes to avoid detrimental effects induced by different stressful factors. Intracellular accumulation of these molecules allows to maintain osmotic balance preventing irreversible dehydration of cells but without any interference with cellular processes (Roessler and Müller 2001; Yancey 2005; Pastor et al. 2013). Compatible solutes can be classified into several structural groups: sugars (trehalose, sucrose), polyols (glycerol, sorbitol, mannitol, α-glucosyl-glycerol, mannosyl-glycerol, and mannosyl-glyceramide), *N*-acetylated diamino acids (like *N*-acetylglutaminylglutamine amide), betaines (such as glycine betaine and derivatives), amino acids (proline, glutamate, glutamine, alanine, ectoine, and hydroxyectoine), and derivatives (Yancey 2005).

Ectoine (ECT) (1,4,5,6-tetrahydro-2-methyl-4-pyrimidine carboxylic acid) is a natural water-binding osmoprotective organic amino acid, which was first isolated from *Ectothiorhodospira halochloris*. This molecule is produced by aerobic, chemoheterotrophic, and halophilic bacteria to survive under extreme conditions (Galinski et al. 1985; Nagata and Wang 2001). Microorganisms such as *Marinococcus* sp. synthesize and accumulate ECT in response to osmotic stress in a hyperosmotic environment (Wei et al. 2011). Production of this amino acid was also reported in bacteria temporarily inhabiting aquatic environments such as *Vibrio cholerae* which adapt to changes in osmolarity (Pflughoeft et al. 2003). ECT is known to increase stability of cell membranes (Harishchandra et al. 2010). Protective effects of ECT were reported to be induced in bacterial membranes, enzymes, and nucleic acids against hyperthermia. ECT was demonstrated to enhance stability of phytase, lactic dehydrogenase (LDH), and phosphofructokinase (PFK), enzymes that are susceptible to heating, urea, freezing and drying, and freeze–thaw treating (Lippert and Galinski 1992; Göller and Galinski 1999; Knapp et al. 1999; Zhang et al. 2006). ECT also induces anti-inflammatory effects in the airways and gastrointestinal tract of mammals (Sydlik et al. 2009; Abdel-Aziz et al. 2013; Unfried et al. 2014). It has been proposed that ECT enhances the hydration of the cell surface by binding water molecules and thereby increasing the mobility of the lipid head groups and fluidizing the lipid layer. The increased fluidity may be advantageous for cell membranes to cope with extreme conditions like high or low temperatures or alteration of osmotic pressure and may accelerate repair mechanisms in some cells (Harishchandra et al. 2010).

*Daphnia magna* is a model animal used in different fields of toxicology. Different compounds have been tested with the use of daphnids, since they possess thin layer of transparent carapace enabling optical measurements of activity of different organs such as heart and thoracic limbs (Villegas-Navarro et al. 2003; Campbell et al. 2004; Penalva-Arana et al. 2011). Although our previous studies showed that this bacterial osmoprotectant possesses low toxicity and induces thermoprotective effects on daphnids (Bownik et al. 2014, 2015), it is still an open question whether this amino acid may induce protective effects in cladocerans exposed to organic solvents such as ETH. Therefore, the purpose of our study was to determine the influence of ECT on *Daphnia magna* subjected to ETH and the following endpoints were determined: immobilisation, swimming velocity, heart rate, thoracic limb movement, catalase activity, and nitric oxide species (NO_*x*_) level.

## Materials and methods

### Culture method

*Daphnia magna* were cultured in a continuous parthenogenetic reproduction for several generations in 17 L tanks with 16 L of aerated Daphnia culture medium on the window ledge in a laboratory under light: dark period of 16h:8 h. *Daphni*a culture medium was prepared following the ASTM standards (American Society of Testing and Materials 1986). The medium was synthetic freshwater (48 mg of NaHCO_3_, 30 mg of CaSO_4_·2H_2_O, 30 mg of MgSO_4_, and 2 mg of KCl per liter of deionized water adjusted to a pH of 7.4), with a temperature of 23 ± 2 °C. The number of cultured daphnids was about 30 animals per liter. The experimental animals were fed once daily with a few drops of *Spirulina* suspension (2 mg/L) per tank and supplemented with tank of 10 mg/L stock suspension of baker’s yeast (100 µL per tank). Feeding was stopped 24 h before the experiments.

Neonates < 24 h old of 2nd–5th clutches were used in the study. Daphnids that were not treated with ECT were maintained in clean medium only.

### Chemicals and experimental design

Ethanol (ETH) (96%) was purchased from POCH (Polskie Odczynniki Chemiczne). Ectoine (ECT) of 99% purity produced by *Halomonas elongata* was purchased from Sigma-Aldrich. Two concentrations: 20 and 60 g/L of ETH alone were selected on the basis of our previous toxicity trials and the toxicological data (Dom et al. 2012). Exposure of daphnids to these two different concentrations of the toxicant alone allowed us to observe different levels of toxic changes after 24 h. After determination of toxic concentrations of ETH alone, we selected appropriate concentrations of ECT to be used in each combination at which we expected possible protective effects. We used the non-toxic concentrations with documented protective effects of ECT from our previous experiments (Bownik et al. 2014, 2015). In each treatment, the experimental animals were exposed to ETH alone at concentrations of 20 and 60 g/L and the following combinations of ETH and various concentrations of ECT: (a) 20 g/L ETH + 5 mg/L ECT, (b) 20 g/L ETH + 10 mg/L ECT, (c) 20 g/L ETH + 25 mg/L ECT, (d) 60 g/L ETH + 5 mg/L ECT, (e) 60 g/L ETH + 10 mg/L ECT, and (f) 60 g/L ETH + 25 mg/L ECT. Each solution was renewed after 24 h. The unexposed animals in medium only were treated as the control.

### Determination of *Daphnia magna* immobilisation

Ten daphnids (in five replicates) were placed in 150 mL glass beakers containing 100 mL of ETH alone (at 20 or 60 g/L) or the appropriate combination of ETH + ECT. Immobilisation of the experimental animals was determined after 6, 24, 48, 72, 96, and 120 h of the exposure. The control daphnids were placed in clean medium only (100 mL).

### Swimming velocity

Swimming velocity of *Daphnia magna* neonates was determined by the method described by Shimizu et al. (2002) with some modifications. Ten animals (in five replicates) were transferred at appropriate times from one the experimental beaker to the observation plastic dish of 55 mm diameter containing 6 mL of the same experimental solution. Swimming velocity of daphnids in each observation dish was video recorded for a minimum of 1 min (with a speed of 30 frames/s) with a digital camera Nikon D3100 mounted on a stand over the dish. Vertical movement of *Daphnia* was negligible because of very small depth of the medium present in the observation dish. All the individuals returned to the experimental beakers for further exposure after the examination. The video file with the recorded trajectories of swimming *Daphnia magna* was analyzed by a frame-by-frame method with Tracker^®^. By clicking with the cursor on *Daphnia* image in separate frames, the program plotted the whole trail left by a single *Daphnia* (interpreted by the program as a mass point) measuring its maximal, minimal, and mean velocity (*v*) expressed in millimeters per second (mm/s). Since the animals moved virtually only in two dimensions, swimming behaviour analysis was based on the trajectory represented by *x-* and *y*-coordinates. The velocities of ten daphnids calculated by software were plotted in the separate graphs which were then superimposed. Since swimming speed was not equal for all individuals in each experimental group and the control, the mean velocity (*v*) of ten daphnids from each experimental group was meant and treated as one result.

### Physiological parameters: heart rate and thoracic limb activity

Physiological parameters were determined by optical measurement of heart rate and thoracic limb movement at appropriate times. A single daphnid was transferred in a 50 µL droplet of appropriate experimental solution to a microscope slide. Movements of the examined animals were limited by cotton wool fibers placed on the slide. The microscopic view of the examined daphnid was recorded for more than 1 min (with a speed of 30 frames/s) with a digital camera Nikon D3100 mounted on a light microscope. The magnification (30–100×) and camera resolution allowed to record the activity of heart and thoracic limbs with a good quality. Video analysis was done with Tracker^®^ software by a frame-by-frame method and counting separate heart contractions during 1 min. Thoracic limb movement was also determined by a frame-by-frame video analysis and counting the separate movements (beats) of the limbs per 1 min.

### Catalase activity

Catalase activity was determined with a spectrophotometric method described by Goth (1991) with its modification to a micromethod. Briefly, living individuals from each experimental and control groups were taken after 6 and 24 h of the exposure, and homogenized in a pestle micro-homogenizer with 200 µL of PBS. The homogenized samples were centrifuged and 100 µL of supernatants were transferred to a 96-well microplate (Nunc) and incubated with 100 µL of 60 µM H_2_O_2_/60 mM sodium potassium buffer at room temperature for 60 s in a 96-well microtiter plate at room temperature. The enzymatic reaction was stopped by the addition of 250 µL of ammonium molybdate (32 mM) and the absorbance of the yellow molybdate/hydrogen peroxide complex was measured with a spectrophotometric microplate reader (Biorad 550) at 415 nm. A mixture of ammonium molybdate and the buffer was treated as blank. All samples were done in triplicate.

### Nitric oxide species (NO_*x*_)

Nitric oxide (NO) has a short half-life; therefore, its derivatives, stable end products of nitric oxide metabolism, were used as a surrogate markers of NO. Briefly, daphnids from the experimental and control group were taken after 6 and 24 h of the exposure and washed in artificial medium, dried on a paper towel, and homogenized in a pestle micro-homogenizer in 200 µL of PBS. The suspension was sonicated with ultrasonic homogenizer (Omni Ruptor 4000) to disrupt the remaining cells and cell membranes. Afterwards, homogenates were centrifuged at 5000*g* for 5 min. After centrifugation, 100 µL of supernatant were taken and placed in wells of a 96-well microplate (Nunc). The wells from each experimental and control groups were done in triplicate. NO_*x*_ level was measured by the Griess reaction (Griess 1879; Smith et al. 2000) by adding 50 µL of 1% sulfanilamide in 5% H_3_PO_4_ to each well and subsequently 50 µL of naphthylethylenediamine dichydrochloride in distilled water. The microplate was incubated at room temperature for 15 min and the absorbance was read with a spectrophotometric microplate reader (Biorad 550) at 550 nm. All samples were done in triplicate.

### Statistical analysis

The results are presented as means ± standard deviation (SD). Data were assessed for homogeneity of variance for ANOVA assumptions. Experimental data were analyzed using ANOVA followed by Tukey’s test to detect differences among means. All analyses were performed using Develve^®^ statistical software. Values were statistically significant when *p* ≤ 0.05.

## Results

### Immobilisation

**Fig. 1:**
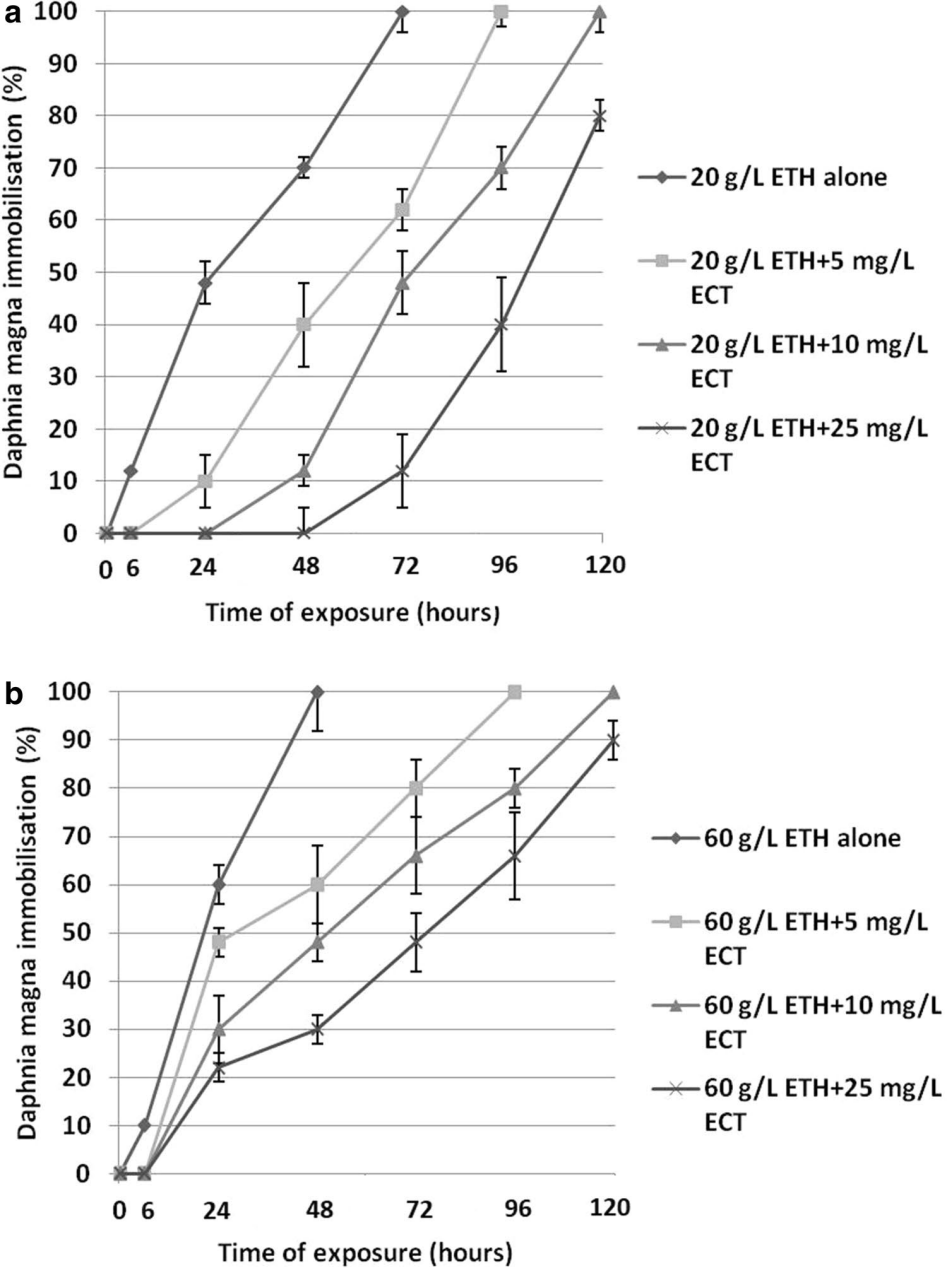
Immobilisation of *Daphnia magna* subjected to 20 g/L (**a**) and 60 g/L (**b**) of ethanol (ETH) alone and in the combinations with various concentrations of ectoine (ECT). The results are presented as means ± SD; *n* = 50

The results are presented in Fig. 1. Exposure to 20 g/L ETH alone induced 70 ± 2% immobilisation of daphnids after 48 h (Fig. 1a). On the other hand, the animals treated with the combinations of 20 g/L ETH with various concentrations of ECT manifested a significant reduction of immobilisation. Moreover, no immobilised daphnids were found after 48 h in the combination of 20 g/L ETH + 25 mg/L ECT. The combinations with lower concentrations of ECT also reduced the percentage of immobilised daphnids; however, the protective effect was less significant.

60 ± 4% and 100% of daphnids exposed to 60 g/L ETH alone were immobilised after 24 and 48 h, respectively (Fig. 1b). However, the microcrustaceans treated with the combinations of ETH with different amounts of ECT showed lower percentage of immobilisation. The most reduced immobilisation (22 ± 3% after 24 h and 30% after 48 h) was observed in the group of daphnids exposed to the mixture of 60 g/L ETH + 25 mg/L ECT. 20% of daphnids from this experimental group were motile even at 120 h of the exposure.

### Swimming velocity

**Fig. 2:**
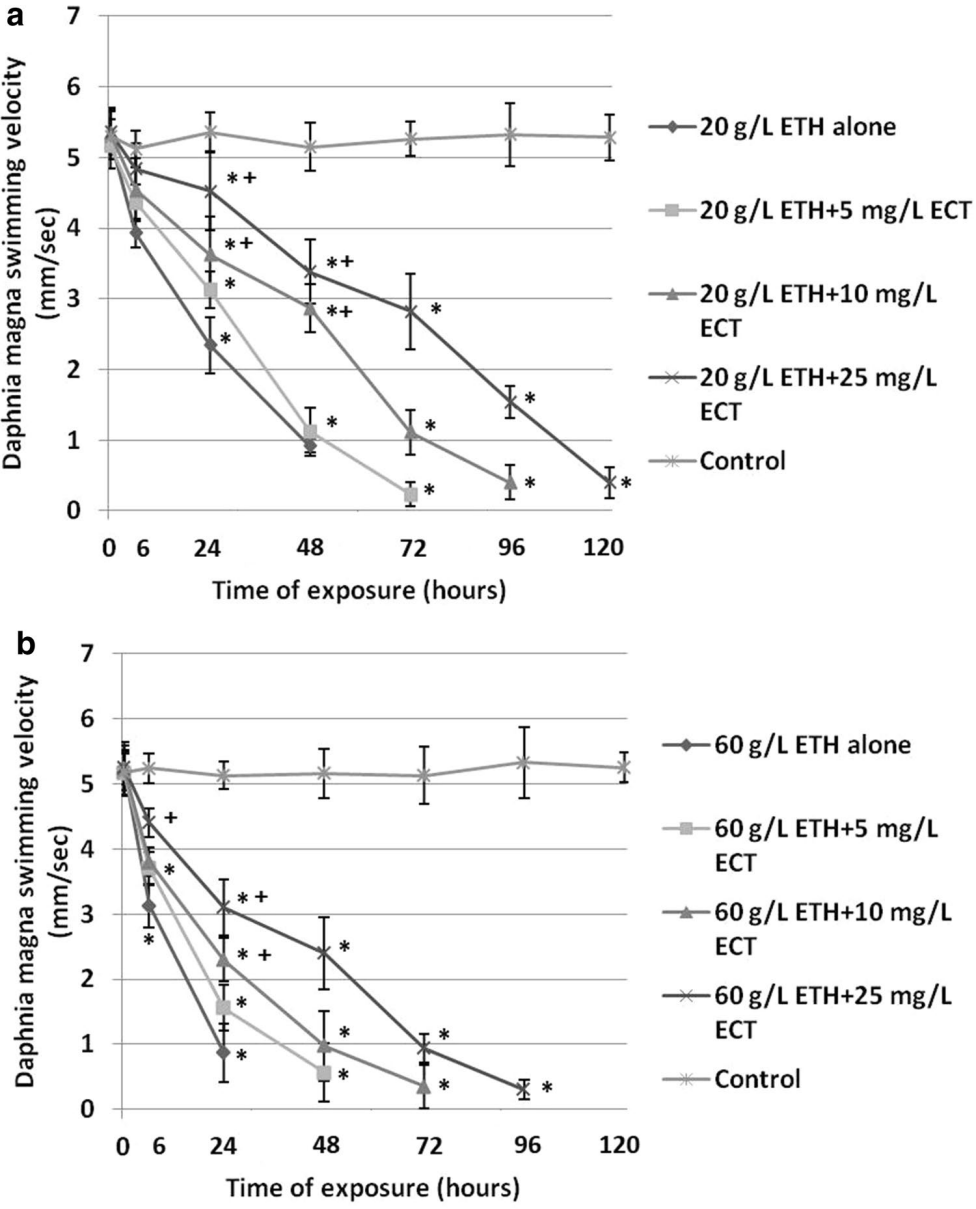
Swimming velocity of *Daphnia magna* subjected to 20 g/L (**a**) and 60 g/L (**b**) of ethanol (ETH) alone and in the combination with various concentrations of ectoine (ECT). The results are presented as means ± SD, *statistical significance when compared to the control; “+” indicates statistical significance between the group with ETH alone and the combinations of ETH + ECT; *n* = 50

The crustaceans exposed to 20 g/L ETH alone manifested a reduced swimming speed to 2.34 ± 0.4 mm/s after 24 h and 0.92 ± 0.1 mm/s after 48 h when compared to the non-treated control (5.36 ± 0.27 mm/s) (Fig. 2a). However, less decreased velocity showed daphnids subjected to the combinations of ETH with various amounts of ECT. The lowest reduction of swimming speed was observed in the animals treated with the combination of 20 g/L ETH + 25 mg/L ECT (4.52 ± 0.55 mm/s after 24 h and 3.38 ± 0.45 mm/s after 48 h).

Exposure of daphnids to 60 g/L ETH alone resulted in a significant decrease of swimming velocity to 0.87 ± 0.45 mm/s after 24 h (Fig. 2b). On the other hand, the animals treated with the combinations of 20 g/L ETH with different concentrations of ECT showed less reduced values. The lowest decrease of swimming velocity was noted in the group exposed to the mixture of 60 g/L ETH + 25 mg/L ECT (3.1 ± 0.43 mm/s after 24 h). Daphnids from this experimental group showed motility even at 96 h of the exposure. Less inhibited values of swimming velocity were also observed in the other combinations of ETH + ECT.

### Heart rate

**Fig. 3:**
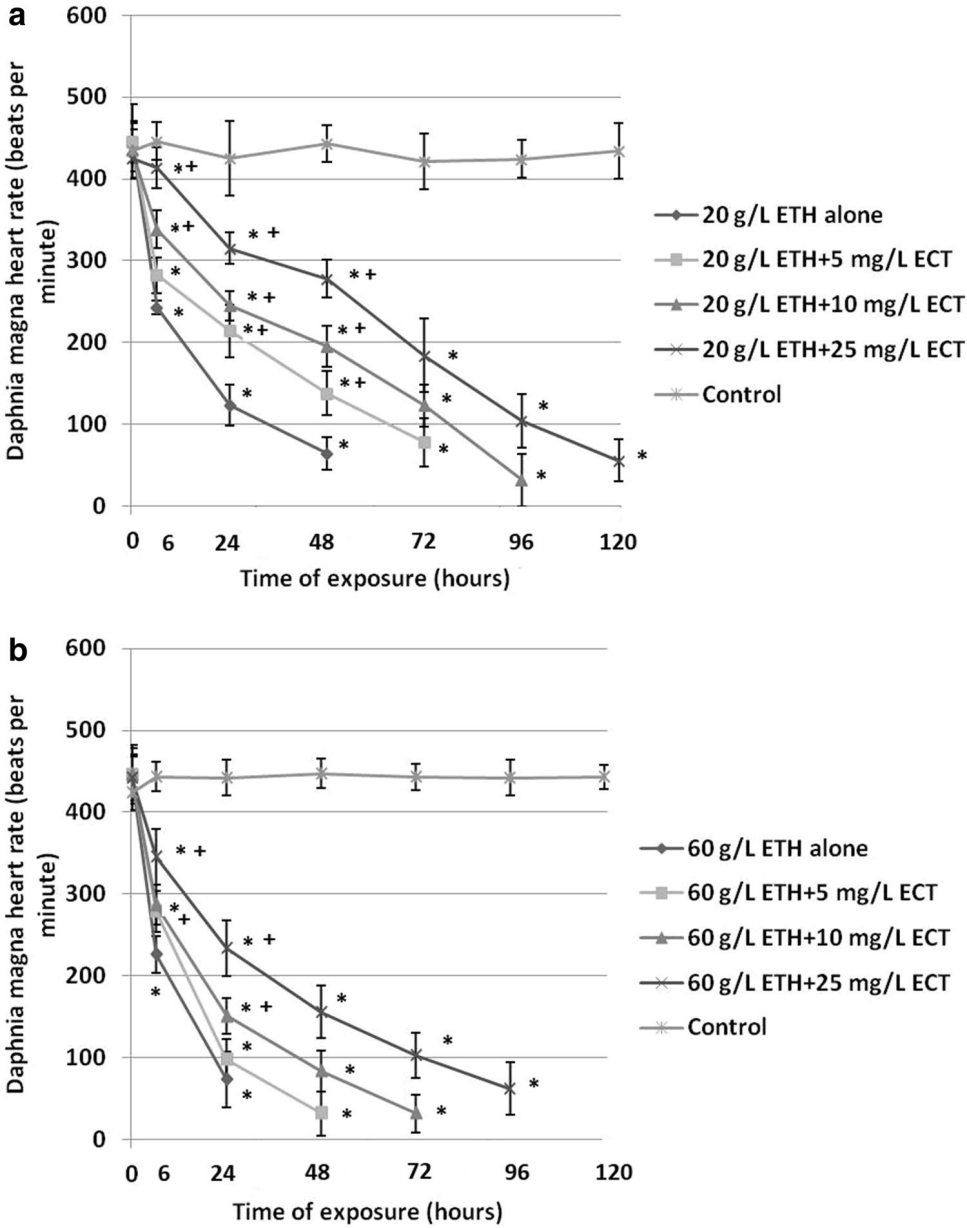
Heart rate of *Daphnia magna* subjected to 20 g/L (**a**) and 60 g/L (**b**) of ethanol (ETH) alone and in the combination with various concentrations of ectoine (ECT). The results are presented as means ± SD, *statistical significance when compared to the control, “+” indicates statistical significance between the group with ETH alone and the combinations of ETH + ECT; *n* = 50

The results are presented in Fig. 3. 24-h exposure of daphnids to 20 g/L ETH alone resulted in a rapid decrease of heart rate to 123 ± 25 bpm after 24 h and 64 ± 20 bpm after 48 h when compared to the control value (445 ± 34 bpm) (Fig. 3a). On the other hand, less decreased heart rate was observed in all the groups of animals treated with the combinations of ETH and various concentrations of ECT. A significantly lower inhibition of heart rate showed daphnids exposed to 20 g/L ETH + 25 mg/L ECT (315 ± 19 bpm after 24 h).

The crustaceans treated with 60 g/L ETH alone showed the most distinct decrease of heart rate (73 ± 34 bpm after 24 h) (Fig. 3b). However, a lower inhibition was found in the groups treated with the combinations of 60 g/L ETH with different amounts of ECT. Very significant attenuation of heart rate decrease showed daphnids exposed to 60 g/L ETH + 25 mg/L (234 ± 34 bpm after 24 h).

### Thoracic limb activity

**Fig. 4:**
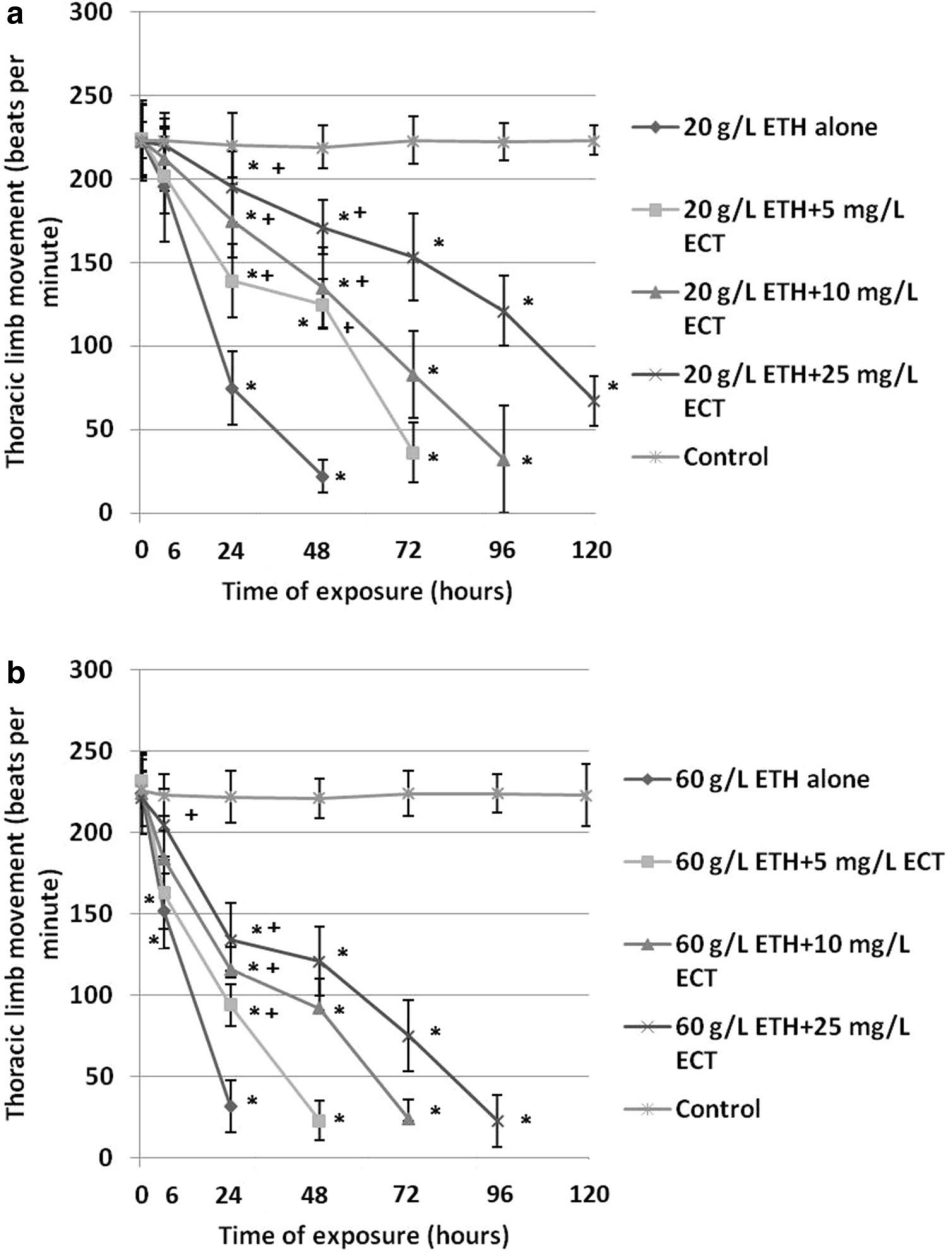
Thoracic limb movement of *Daphnia magna* subjected to 20 g/L (**a**) and 60 g/L (**b**) of ethanol (ETH) alone and in the combination with various concentrations of ectoine (ECT). The results are presented as means ± SD, *statistical significance when compared to the control, “+” indicates statistical significance between the group with ETH alone and the combinations of ETH + ECT; *n* = 50

Daphnids exposed to 20 g/L ETH alone manifested a distinct inhibition of thoracic limb activity (75 ± 22 bpm after 24 h and 22 ± 10 bpm after 48 h) (Fig. 4a) when compared to the control (224 ± 24 bpm). However, a lower decrease was found in the animals treated with the combinations of ETH with various amounts of ECT. A significant alleviation of thoracic limb movement decrease showed daphnids exposed to 20 g/L ETH + 25 mg/L ECT (195 ± 21 bpm after 24 h and 171 ± 16 bpm after 48 h).

Exposure to 60 g/L ETH alone resulted in the most distinct reduction of thoracic limb movement (32 ± 16 bpm after 24 h) (Fig. 4b). On the other hand, the animals treated with the combinations of ETH + ECT showed a lower level of the limb activity inhibition. A significant attenuating effect was noted in the mixture of 60 g/L ETH + 25 mg/L ECT (134 ± 23 bpm after 24 h).

### Catalase activity

**Fig. 5:**
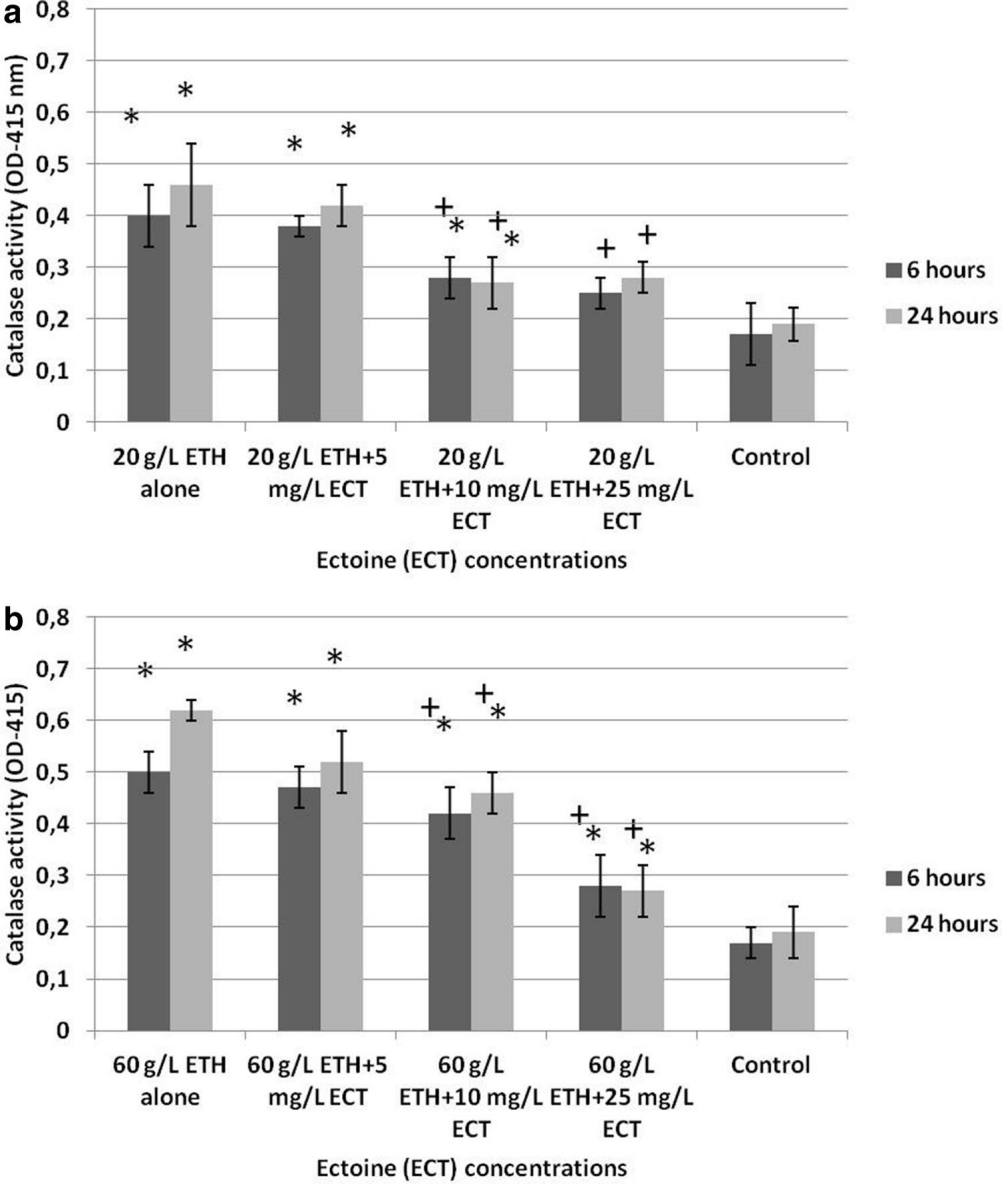
Catalase activity of *Daphnia magna* subjected for 6 and 24 h to 20 g/L (**a**) and 60 g/L (**b**) of ethanol (ETH) alone and the combination with various concentrations of ectoine (ECT). The results are presented as means ± SD. OD—optical density. *Statistical significance between the experimental and control groups *p* ≤ 0.05, “+” indicates statistical significance between the group with ETH alone and the combinations of ETH + ECT

Increase of catalase activity was observed in the group of daphnids exposed to 20 g/L ETH alone (OD = 0.4 ± 0.06 after 6 h and 0.46 ± 0.04 after 24 h) (Fig. 5a) in comparison to the control (OD = 0.17 ± 0.03 after 6 h and 0.19 ± 0.032 after 24 h). On the other hand, a less pronounced increase of activity was observed in the crustaceans treated with the combinations of ETH + ECT. The most significant attenuation of the activity was manifested in daphnids exposed to 20 g/L ETH + 25 mg/L ECT (OD = 0.17 ± 0.03 after 6 h and 0.19 ± 0.05 after 12 h).

Treatment of daphnids with 60 g/L ETH alone resulted in the most distinct increase of catalase activity (OD = 0.5 ± 0.04 after 6 h and 0.62 ± 0.02 after 24 h) (Fig. 5b). However, exposure with the combinations of 60 g/L ETH with various concentrations of ECT induced a lower stimulation of the enzyme activity. A significant alleviation of the increase was observed in the group exposed to 60 g/L ETH + 25 mg/L ECT (OD = 0.28 ± 0.06 after 6 h and 0.27 ± 0.05 after 24 h).

### NO_*x*_ level

**Fig. 6:**
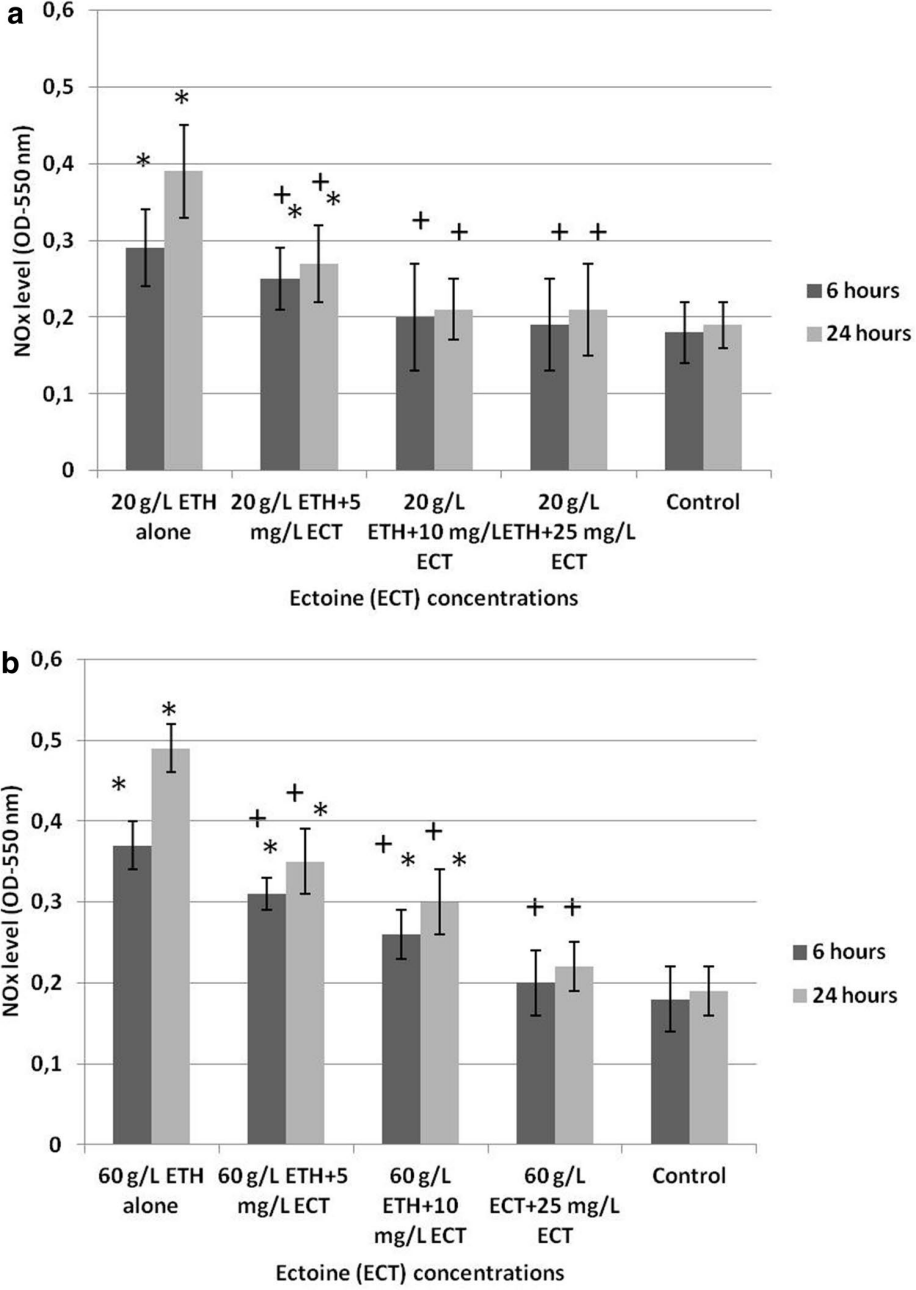
NO_*x*_ level of *Daphnia magna* subjected for 6 and 24 h to 20 g/L (**a**) and 60 g/L (**b**) of ethanol (ETH) alone and in the combinations with various concentrations of ectoine (ECT). The results are presented as means ± SD. OD—optical density. *Statistical significance between the experimental and control groups *p* ≤ 0.05, “+” indicates statistical significance between the group with ETH alone and the combinations of ETH + ECT

Daphnids exposed to 20 g/L ETH alone showed an increased level of NO_*x*_ species (OD = 0.29 ± 0.05 after 6 h and 0.39 ± 0.06 after 24 h) when compared to the non-treated control (0.18 ± 0.04 after 6 h and 0.19 ± 0.03 after 24 h) (Fig. 6a). In contrast, exposure of the animals to the combinations of ETH + ECT attenuated the increase of NO_*x*_ level with a very distinct effect noted at 20 g/L ETH + 25 mg/L ECT (OD = 0.19 ± 0.06 after 6 h and 0.21 ± 0.06 after 24 h).

Treatment of the animals with 60 g/L ETH alone resulted in the most pronounced increase of NO_*x*_ level (OD = 0.37 ± 0.03 after 6 h and 0.49 ± 0.03 after 24 h). Daphnids exposed to the combinations of 60 g/L ETH with various amounts of ECT showed a lower stimulation of NO_*x*_ production. The most significant alleviation was seen in the combination of 60 g/L ETH + 25 mg/L ECT (OD = 0.2 ± 0.04 after 6 h and 0.22 ± 0.33 after 24 h).

## Discussion

Although studies on acute toxicity of pure ETH were previously performed in *Daphnia magna* (Takahashi et al. 1987; Lilius et al. 1995; Dom et al. 2012), very few results are available on its effects on behavioural and biochemical parameters in cladocerans. Some studies with daphnids were performed in systems in which ETH was used in mixtures with different chemicals to determine a possibility of interactions. For example, results obtained by Zhang et al. (2003) in *Daphnia magna* indicated the increased toxicity of 4-nonylphenol when it is dissolved in ethanol. To our best knowledge, this is the first study showing the protective effects of ECT in daphnids subjected to toxic concentrations of ETH. Here, we demonstrate that ECT alleviated the toxic changes in ETH-treated daphnids on the behavioural, physiological, and biochemical levels.

### Immobilisation

Some reports indicate that treatment of *Daphnia magna* with high or very high concentrations of ETH induces rapid immobilisation or death (Takahashi et al. 1987; Lilius et al. 1995; McKenzie et al. 1992). The present study revealed that exposure to ETH alone caused a concentration-dependent immobilisation which was probably associated with the neurotoxic action of ETH. Currently, no data are available on the metabolic pathways of ETH in daphnids. The generally accepted mechanism of neurotoxic effects induced by ETH in vertebrates is related to its interaction with glutaminergic system (Tsai and Coyle 1998). Metabolism of ETH may also lead to the formation of free radicals inducing oxidative stress (Zima et al. 2001; Comporti et al. 2010). The latter scenario may contribute to ETH toxicity in daphnids, since our results showed that catalase activity and NO_*x*_ level correlated with the level of daphnid immobilisation. The present study also demonstrated that exposure of daphnids to the combinations of ETH + ECT manifested a longer time until immobilisation in comparison to the animals treated with ETH alone. The protective effect of the osmoprotectant was more distinct animals exposed to the combinations of lower concentration of ETH and higher amounts of ECT. The group of daphnids with the longest times until immobilisation also showed attenuated toxic changes of biochemical biomarkers of oxidative stress which suggests that the reduced immobilisation was, at least partially, due to antioxidative action of the osmoprotectant.

### Swimming velocity

Swimming performance is a sensitive behavioural biomarker in aquatic animals and a commonly used endpoint in ecotoxicological studies reflecting even slight detrimental effects in neuromuscular functions of an organism (Oliveira et al. 2012; Silva et al. 2013). The present study revealed that ETH alone decreased swimming velocity of daphnids in a concentration-dependent manner. The decreased swimming velocity in daphnids may be associated with its neurotoxic action. This compound is known to depress locomotor activity in various animal species (Matchett and Erickson 1977). On the other hand, we noted that the animals exposed to the combinations of ETH with various concentrations of ECT showed less depressed locomotor activity which may be a result of neuroprotective effects of ECT. The mechanisms of neuroprotection caused by ECT are not known; however, they may be associated with its osmoprotective effects resulting from increased stabilization of cell membrane structure (Harishchandra et al. 2010). Another compatible solute, taurine was also shown to alleviate toxic effects of ethanol in the nervous system of animals and humans (Olive 2002). Our previous study demonstrated that the decrease of swimming velocity was attenuated by ECT in daphnids exposed to higher temperature (Bownik et al. 2014). It suggests that ECT may induce protective effects on nervous system of animals exposed to different kinds of stressors.

### Heart rate and thoracic limb activity

Alteration of heart rate in daphnids is a well-known effect induced by various compounds (Villegas-Navarro et al. 2003; Campbell et al. 2004). Transparency of the carapax allows to determine possible changes of this physiological parameter with non-invasive methods such as light microscopy. The present study revealed that both concentrations of ETH alone rapidly induced bradycardia. The mechanism of this effect in daphnids is not known; however, it may be associated with ETH-induced changes of neurotransmitter release or toxic changes in their receptors. Such interactions were shown in studies performed on vertebrates (Wu et al. 1994; Cardoso et al. 1999). It is also possible that ETH directly affects the heart muscle cells as it was shown in mammals (Thomas et al. 1994). We also showed that daphnids exposed to the combinations of ETH with ECT showed a significant attenuation of bradycardia. The alleviating effects were concentration-dependent, but the mechanisms are not known; however, it may be speculated that they may be linked to antioxidative effects of the amino acid and thereby stabilization of neurotransmitter receptors or osmoprotective action on the neurons and cardiomyocytes. Interestingly, ECT alone was found to modulate heart rate of daphnids. Lower concentrations of the amino acid induce slight tachycardia; however, its higher amounts cause bradycardia (Bownik et al. 2015).

Thoracic limbs play an important role in the process of ventilation reflecting the metabolic activity of *Daphnia magna*. Thoracic limb movement may be altered by various agents; therefore, their activity is considered to be a biomarker of environmental stress (Pirow et al. 1999; Penalva-Arana et al. 2011). We noted that 24-h exposure to both concentrations of ETH alone induced a concentration-dependent decrease of the limb activity which may be a result of neuromuscular depression induced by ETH. On the other hand, a significantly lower reduction of the limb activity of daphnids treated with the combinations of ETH and various concentrations of ECT is probably a consequence of neuroprotective action. Modulatory effects of ECT alone on thoracic limb activity were reported in our previous experiments (Bownik et al. 2015). This amino acid was found to stimulate the activity of thoracic limbs at lower concentrations; however, its higher amounts induced opposite effects. Results of our another study indicated that ECT induces protective activity on thoracic limb movement in heat-stressed daphnids (Bownik et al. 2014).

### Catalase activity

There are a number of studies that reported changes of catalase activity induced by different chemicals in aquatic animals (Ahmad et al. 2005; Jemec et al. 2007, 2010; Sarkar et al. 2014). Catalase was reported to be involved in the oxidation of ETH in mammals with a species-dependent difference in its activity (Hawkins and Kalant 1972; Oshino et al. 1973). Alterations of this enzyme activity also occur in daphnids exposed to various toxic compounds and it was suggested to be a biomarker of oxidative stress in *Daphnia magna* (Kim et al. 2009, 2010; Fan et al. 2012). Results obtained in the present study indicate that both concentrations of ETH induced increase of catalase activity; however, more distinct effect was seen at its higher concentration. The activity of the enzyme was also more stimulated with increasing time of the exposure. On the other hand, daphnids treated with the combinations of ETH with ECT manifested less stimulated activity of catalase. It suggests that ECT may induce protective, presumably antioxidative effects which prevented the stimulation of the enzyme activity. Our previous study indicating thermoprotective effect of ECT showed that high temperature induced increase of catalase activity in daphnids that were not treated with ECT; however, lower stimulation of its activity was observed in ECT-treated animals (Bownik et al. 2014). In another study, we found alteration of this enzyme in daphnids exposed to ECT alone. Higher concentration of ECT alone turned out to slightly stimulate catalase activity (Bownik et al. 2015).

### NO_*x*_ level

NO is a signalling molecule which has many various roles in animal organisms (Regulski and Tully 1995; Demenge and Ribuot 2001). Antioxidant capacity may occur at its lower concentrations; however, its high levels may be associated with oxidative damage (Borniquel et al. 2006). Since NO is unstable, its derivatives (nitrites and nitrates) are usually evaluated. We found that both concentrations of ETH alone induced a significant increase of NO_*x*_ level. The values of NO_*x*_ were elevated with the increasing time of the exposure. However, daphnids exposed to the combinations of ETH with various amounts of ECT turned out to alleviate the increased NO_*x*_ level. Although mechanisms of protective action of ECT are not known, they may be related to the increased resistance to oxidation due to stabilization of structural organization of the cell membranes (Harishchandra et al. 2010). Therefore, alleviation of ethanol-induced toxicity by the amino acid may be related to decreased permeability of cell membrane for alcohol and, thus, its lower amounts entering the cells and hemolymph.

Taken together, we found that ETH alone induces immobilisation and toxic alterations of behavioural, physiological, and biochemical parameters of *Daphnia magna*. Results of biochemical endpoints indicate that one of the possible mechanisms of its toxicity in these invertebrates may be oxidative stress. We also showed that ECT alleviated toxic effects of ETH. Our findings suggest that one of the protective mechanisms of this osmoprotectant may be the reduction of oxidative stress.
